# Does pre-operative psychological distress affect patient satisfaction after primary total hip arthroplasty?

**DOI:** 10.1186/1471-2474-12-122

**Published:** 2011-06-01

**Authors:** Munier Hossain, Daniel J Parfitt, David J Beard, Clare Darrah, John Nolan, David W Murray, Glynne Andrew

**Affiliations:** 1 Department of Trauma and Orthopaedics, Ysbyty Gwynedd, Bangor LL57 2PW, UK; 2 Nuffield Department of Orthopaedic Surgery, Nuffield Orthopaedic Centre NHS Trust Headington, Oxford OX3 7LD, UK; 3 Norfolk and Norwich University Hospital Norwich, NR4 7UY, UK

## Abstract

**Background:**

There are concerns that pre-operative psychological distress might be associated with reduced patient satisfaction after total hip replacement (THR).

**Methods:**

We investigated this in a multi-centre prospective study between January 1999 and January 2002. We dichotomised the patients into the mentally distressed (MHS ≤ 56) and the not mentally distressed (MHS > 56) groups based on their pre-operative Mental Health Score (MHS) of SF36.

**Results:**

448 patients (340 not distressed and 108 distressed) completed the patient satisfaction survey. Patient satisfaction rate at five year was 96.66% (415/448). There was no difference in patient satisfaction or willingness to have the surgery between the two groups. None of pre-operative variables predicted five year patient satisfaction in logistic regression.

**Conclusions:**

Patient satisfaction after surgery may not be adversely affected by pre-operative psychological distress.

## Background

Total Hip Replacement (THR) is recognised as a very successful surgical intervention with high patient satisfaction [1]. However, it is estimated that a significant proportion of the patients may remain unsatisfied following THR [2]. Researchers have analysed various preoperative patient characteristics in an attempt to identify the best candidates for surgery and to identify the factors that are likely to predict poor patient satisfaction postoperatively [3-7]. The effect of patients' preoperative mental health on the outcome of joint replacement surgery has become an area of interest and has received some attention [8,9]. Researchers have shown that patients with preoperative mental distress are less likely to benefit after knee arthroplasty but the effect on outcome is less clear with THR [2,8-10].

This prospective study investigated what effect preoperative mental health-assessed as psychological distress- had on patient satisfaction after THR. Our null hypothesis was that there was no difference in patient satisfaction after THR between patients who reported mental distress before surgery and those who did not.

## Methods

The Exeter Primary Outcome Study (EPOS) was a multi centre prospective cohort study of outcome after primary THR. Patients were consecutively recruited between January 1999 and January 2002 at seven centres across England and Scotland. Patients underwent THR using a cemented Exeter femoral stem component (Stryker Howmedica Osteonics, Mahwah, New Jersey). A variety of cemented and uncemented acetabular components were used. Patients were included if they were undergoing primary hip arthroplasty with an Exeter cemented femoral stem and were willing and able to give consent to participate in the study. The study conformed to the Helsinki declaration and to local legislation. North Western Multiple Centre Research Ethics Committee and the local research ethics committees in all the participating centres gave ethical approval for conducting the study. Four centres collected data for SF-36 and patient satisfaction survey. We prospectively recorded patients' demographic and clinical status. We also recorded SF-36 score before and after surgery. We obtained the Medical Outcome Survey Short Form 36 (SF-36) score to assess physical, mental and functional status of the patients. The SF-36 is a widely used generic instrument designed to measure patients' health related quality of life. Higher score in SF36 for a domain means better physical or mental state (0-100 range). We used the mental health (MHS) score of SF-36 as a validated marker of mental distress. Patients were dichotomised into a group classed as self-reported psychological distress with a mental health score of fifty-six or less (MHS ≤56) and those not psychologically distressed (MHS > 56) based on their pre-operative MHS. The cut-off value of 56 has been suggested by the European Mindful Working Party [11]. At five year follow-up we also invited the patients to complete a patient satisfaction survey. 448/819 completed the patient satisfaction survey.

The patient satisfaction survey was a seven item questionnaire that included questions on pain relief, improvement in ability to do house or garden work, improvement in ability to do recreational activities, overall satisfaction, improvement of quality of life, residual concerns about the operated hip, and whether patients would be prepared to have the same procedure again. The first four questions were graded on a four point likert scale (1 = very satisfied, 2 = somewhat satisfied, 3 = somewhat dissatisfied, 4 = very dissatisfied). Quality of life improvement was indicated by a six point likert scale (1 = more than imagined, 2 = great improvement, 3 = moderate, 4 = little, 5 = none, 6 = worse). Patient concern was indicated by a dichotomous reply (yes/no). The satisfaction survey is reproduced in detail in table 1. Similar questionnaires have been used by various published studies to indicate patient satisfaction following joint replacement [12,13]. Besides, the single question alone (overall satisfaction) has been well validated as a correct indicator of patient satisfaction [14]. Patients were followed up in the outpatient clinic where they completed the patient satisfaction and SF-36 questionnaires. We retrospectively reviewed the database to analyse the relationship between pre-operative psychological distress and post-operative patient satisfaction for the 448 patients (340 not distressed and 108 distressed) who had complete data for pre-operative psychological distress and five year patient satisfaction.

**Table 1:** Patient satisfaction survey

**How satisfied are you with your hip replacement**

1. For relieving your pain?
(1 = very satisfied, 2 = somewhat satisfied, 3 = somewhat dissatisfied, 4 = very dissatisfied).
2. For improving your ability to do housework or garden work?
(1 = very satisfied, 2 = somewhat satisfied, 3 = somewhat dissatisfied, 4 = very dissatisfied).
3. For improving your ability to do recreational activities?
(1 = very satisfied, 2 = somewhat satisfied, 3 = somewhat dissatisfied, 4 = very dissatisfied).
4. How satisfied are you with the results of your hip replacement surgery?
(1 = very satisfied, 2 = somewhat satisfied, 3 = somewhat dissatisfied, 4 = very dissatisfied).
5. How much did the hip replacement surgery improve the quality of life?
(1 = more improvement than I ever dreamed of, 2 = a great improvement, 3 = a moderate improvement, 4 = a little improvement, 5 = no improvement at all, 6 = the quality of my life is worse)
6. Knowing how well you have done following your hip replacement surgery, would you have the same surgery again?
1 = Yes, 2 = no, 3 = unsure
7. Do you have any worries or concerns about your hip? 1 = Yes, 2 = no

All eligible patients were invited to participate in the study. Patient recruitment varied between the centres but was between 80%-90% of eligible patients. The geographical area covered by the participating hospitals was wide and included both university teaching and district general hospitals that included urban as well as rural locations and represented both affluent as well as inner city suburbs. The catchment area of the four combined units included over a million people.

### Statistics

We used SPSS 16.0 (SPSS Inc, Illinois, USA) statistical software for statistical analysis. The Mann-Whitney test was used for non-parametric data and t test for parametric data. Differences in proportions were tested using the χ^2 ^test or, where necessary, Fisher's exact test. In all analyses we used the two tailed test. We considered results significant at *p *< 0.05. We performed binary logistic regression to identify the variables predictive of patient satisfaction at five years after surgery. We dichotomised the four point patient satisfaction score into the satisfied ("very satisfied" or "somewhat satisfied") or the not satisfied ("somewhat dissatisfied "or "very dissatisfied") groups. The dichotomised variable was introduced as the dependent variable. Independent variables were the various baseline characteristics and pre-operative SF36 domain scores. Variables were initially tested for significance using univariate analysis and significant variables introduced in a multivariate analysis to test for significance and to identify adjusted odds ratios. Different authors have used different cut-off MHS values to define mental distress [8]. We therefore performed a sensitivity analysis with a cut-off MHS value of 50 to test the robustness of our conclusion. We also stratified the results of the patient satisfaction survey according to gender.

## Results

The patients' demographics and pre-operative details are shown in Table 1. Females were significantly more common in the psychologically distressed group. The distressed group also weighed less and were shorter in height compared to the non - distressed group. The group reporting pre-operative psychological distress had significantly worse pre-operative SF-36 scores in all domains (Table 2).

**Table 2:** Patient baseline characteristics with mean values and range in brackets

Patient characteristics	Non distressed (n = 340)	Distressed (n = 108)	*p *value
Gender: M:F	147:192	33:75	0.018
Height: ( cm)	167.95 ( 146-189)	165.90 (145-185)	0.047
Weight ( kg)	78.01 ( 48-154)	74.67 ( 44-126)	0.058
Age	64.37 (29-90)	64.70 (23-85)	0.772
Affected hip	L 163, R 167	L 38 R 70	0.026
Occupation	Unemployed/retired 218 professional 46 other 66	Unemployed/retired 76 housewife 12 other 20	0.040
Diagnosis	Primary OA 271 Secondary OA 28 Other 41	Primary OA 90 Secondary arthritis 6 Other 12	0.080
Co-existent disease	252	90	0.052
Corticosteroid use	47	9	0.693
NSAID	163	53	1.000
Anticoagulant use	39	21	0.054
Charnley category	A 168, B 62, C 90	A 50, B19, C 35	0.559
MHS	79 (60-100)	43 ( 0-56)	0.000
General health	71 (20-100)	59 (10-97)	0.000
Bodily pain	30 ( 0-100)	18 (0-94)	0.006
Physical functioning	21 (0-90)	13 (0-95)	0.002
Social functioning	50 (0-100)	26 (0-100)	0.000
Vitality	44(0-95)	26 (0-70)	0.001
Role emotional	59 ( 0-100)	15 (0-100)	0.000
Role physical	11 (0-100)	5 (0-100)	0.022
Surgical approach	Posterior 79, lateral 160	Posterior 31, lateral 50	NA

### Patient satisfaction

Overall patient satisfaction was high with 3.4% of patients (15/448) remaining "somewhat dissatisfied" or "very dissatisfied" at five years after surgery (Table 3). The patients not reporting pre-operative psychological distress reported significantly lower level of pain in the patient satisfaction questionnaire (p = 0.026), were more able to do recreational activities (p = 0.039) and were less concerned about the operated hip (p = 0.007) compared to the distressed group. However, this did not seem to affect the overall satisfaction (p = 0.410), ability to do housework (p = 0.063), perception of improvement in quality of life (p = 0.349) or the willingness to have the surgery again (p = 0.219) between the two groups at five year following surgery. Gender, primary diagnosis and various domains of SF-36 score were all significantly independently predictive of five year patient satisfaction. However, when the effects of the significant variables were adjusted for in a multivariate analysis their effects were no longer significant and none of the variables were predictive of patient satisfaction.

**Table 3:** Patient satisfaction survey

Overall satisfaction	Very satisfied	Somewhat satisfied	Somewhat dissatisfied	Very dissatisfied	Total
No distress	280(82.4%)	50(14.7%)	8(2.4%)	2(0.6%)	340 (100%)
Distress	81(75.0%)	22(20.4%)	4(3.7%)	1(0.9%)	108 (100%)
Total	361 (80.6%)	72 (16.1%)	12 (2.7%)	3 (0.7%)	448 (100%)
Pain relief	Very Satisfied	Somewhat Satisfied	Somewhat Dissatisfied	Very Dissatisfied	Total
No distress	281(83.1%)	46(13.6%)	10(3.0%)	1(0.3%)	338 (100%)
Distress	79(73.1%)	22(20.4%)	4(3.7%)	3(2.8%)	108 (100%)
Total	360 (80.7%)	68 (15.2%)	14 (3.1%)	4 (0.9%)	446 (100%)
Any concerns regarding operated hip	Yes	No			Total
No distress	66(19.5%)	273(80.5%)			339 (100%)
Distress	34(32.4%)	71(67.6%)			105 (100%)
Total	100 (22.5%)	344 (77.5%)			444(100%)
Have same surgery again?	Yes	No	Unsure		Total
No distress	296(87.3%)	9(2.7%)	34(10.0%)		339(100%)
Distress	88(81.5%)	6(5.6%)	14(13.0%)		108 (100%)
Total	384 (85.9%)	15 (3.4%)	48 (10.7%)		447 (100%)
Quality of life improvement	Great or more than imagined	Moderate	Little	None or worse	Total
No distress	296(87.3%)	32(9.4%)	5(1.5%)	6 (1.8%)	339(100%)
Distress	85(78.7%)	16(14.8%)	4(3.7%)	3 (2.8%)	108 (100%)
Total	381 (85.3%)	48 (10.7%)	9(2.0%)	9 (2.0%)	447 (100%)
Ability to do house/gardenwork	Very satisfied	Somewhat satisfied	Somewhat dissatisfied	Very dissatisfied	Total
No distress	211(63.7%)	95(28.7%)	17(5.1%)	8(2.4%)	331 (100%)
Distress	42(43.3%)	34(39.1%)	7(8.0%)	4(4.6%)	87 (100%)
Total	253 (60.5%)	129 (30.9%)	24(5.7%)	12 (2.9%)	418 (100%)
Ability to do recreational activities	Very Satisfied	Somewhat Satisfied	Somewhat Dissatisfied	Very Dissatisfied	Total
No distress	187(56.5%)	105(31.7%)	27(8.2%)	12(3.6%)	331 (100%)
Distress	35(40.7%)	33(38.4%)	12(14.0%)	6(7.0%)	86(100%)
Total	222 (53.2%)	138(33.1%)	39 (9.4%)	18 (4.3%)	417 (100%)

### Effect of possible confounders

We compared the incidence of post-operative complications or further hip surgery up to five years following the index procedure to assess if postoperative complications or further hip surgery might have acted as a confounding factor that affected patient satisfaction. There was no significant difference in major postoperative complications (p = 0.891) or further hip surgery (p = 0.969) between the two groups of patients (Table 4).

**Table 4:** Post-operative complications and further hip surgery

Post-operative complications	Very satisfied	Somewhat satisfied	Somewhat dissatisfied	Very dissatisfied	Total
No	301(81.4%)	58(15.7%)	10(2.7%)	1(0.3%)	370 (100%)
Yes	4(77.8%)	10(18.5%)	2(3.7%)	0(0.0%)	54 (100%)
Total	361 (80.9%)	72 (16.0%)	12 (2.8%)	1 (0.2%)	424 (100%)
**Further hip surgery**	Very Satisfied	Somewhat Satisfied	Somewhat Dissatisfied	Very Dissatisfied	Total
No	337(80.8%)	67(16.1%)	12(2.9%)	1(0.2%)	417 (100%)
Yes	6(85.7%)	1(14.3%)	0(0.0%)	0(0.0%)	7 (100%)
Total	343 (80.9%)	68 (16.0%)	12 (2.8%)	1 (0.2%)	444 (100%)

### Sensitivity analysis

Sensitivity analysis with a cut-off MHS value of 50 did not affect the results of patient satisfaction.

We also stratified the patient satisfaction survey according to gender. As previously noted, patients reporting pre-operative psychological distress in both genders reported significantly higher level of pain (male p = 0.007, female p = 0.025). The pattern of patient satisfaction with regard to patients' ability to do housework (male p = 0.671, female p = 0.238), ability to do recreational activities (male p = 0.609, female p = 0.151), improvement in quality of life (male p = 0.567, female p = 0.710) and in willingness to have the surgery again (male p = 0.612, female p = 0.399) was the same between the sexes and not significantly different for pre-operative psychological distress. However, unlike the previously observed trend, overall satisfaction differed between the sexes. Men reporting pre-operative psychological distress were significantly more likely to remain overall unsatisfied after surgery (p = 0.016) whereas women were not (p = 0.869. Similarly, men reporting pre-operative psychological distress were highly likely to maintain further worry or concern regarding the operated hip (p = 0.001) whereas women were not (p = 0.227).

## Discussion

Our results indicate that women are more likely to report pre-operative mental distress than men and the mentally distressed group have worse absolute values for all domains of SF-36 score. However, even though patients with pre-operative mental distress report somewhat higher pain and may have more concerns with their operated hip, five year patient satisfaction following THR is very high and is not affected by patients' pre-operative mental distress. Men who reported pre-operative psychological distress were more likely to remain less overall satisfied and less satisfied with pain relief. There appears to be accumulating evidence that patient satisfaction after surgery is multifactorial and complicated. Our results suggest that it may not be possible to predict patient satisfaction at five years after surgery by considering the pre-operative patient variables in isolation. It is intriguing that although the non distressed group reported significantly less pain this did not appear to affect patient satisfaction. Pain relief is known to be an important predictor of post operative satisfaction. On closer scrutiny the difference in pain between the two groups appear minimal (96.7% satisfaction with pain in no distress group compared to 93.5% satisfaction with pain in the distressed group). However, it is notable that we found no significant difference in willingness to go through with surgery again between the two groups. Previously researchers have reported that willingness to go through with surgery again validated a clinically important difference in WOMAC scoring [15]. This would suggest that even though statistically significant, the clinical significance of difference in pain between the two groups may be low and as such did not affect overall patient satisfaction.

The clinical significance of difference in patient satisfaction when stratified for gender remains uncertain and needs to be further investigated. We are not aware of any previous study that has indicated this aspect of patient satisfaction. Previous studies had indicated that gender in isolation does not predict patient satisfaction [13]. It is notable that men reporting pre-operative psychological distress not only remained less satisfied after surgery they were also less satisfied with pain compared to women and remained more concerned after surgery even though there was no difference in reported satisfaction with household or recreational activities. We speculate that this difference might be due to difference in expectation of pain relief between the two groups. Indeed previous reports have indicated that most patients expect improvement in pain and satisfaction is related to patients' previous expectation [13]. Our results also agree with a previous report that post-operative functional capacity does not necessarily correlate with patient expectation [16].

Because of variation in outcome following joint replacement [17], some quarters have expressed reservation about operating on patients who are likely to have poor response [9]. Various studies have therefore attempted to identify possible poor responders following surgery [9,18]. However, it should also be borne in mind that results from large cohort studies may not be applicable at an individual level.

A degenerative joint is a source of chronic pain and physical dysfunction and both of these factors have known association with impaired mental health [19-21]. There are varying reports about the effect of emotional distress or illness on outcomes after joint arthroplasty with reports suggesting both reduced health gain [22-25] and substantial improvement [8]. Although the volume of research on knee replacement is substantial very few researchers have addressed THR patients. A recent study suggested that caution and possibly additional treatment should be employed for patients with mental distress awaiting THR because of the risk of dissatisfaction [9]. Another small study previously contended that even though the health benefit gained by them may be no less, distressed patients reported less absolute WOMAC score pre and postoperatively after THR [10].

We do not know how representative our study population is of the general population undergoing or awaiting THR. Because this is a report from a few self-selected centres it is possible that selection or referral bias might have affected the study population. We also did not record patient expectation. Some have claimed that patient satisfaction after surgery is closely related to their expectation [25].

## Conclusions

The current study demonstrates that five year patient satisfaction after THR is very high and although patients with pre-operative mental distress report less pain relief they remain no less satisfied than those without any mental distress. Willingness to experience the procedure again or the perception of improvement in quality of life is similar in both groups of patients. Patients with pre-operative psychological distress are no more likely to be poor responders after surgery than those not distressed.

## Competing interests

this study was financed by Stryker, UK.

## Authors' contributions

DB, CD, DM, JN and GA conceived and designed the study. CD, MH and DP gathered the data, MH, DP, DB, DM and GA analysed the data, MH, DP and GA wrote the initial draft. DB, DM and GA ensured the accuracy of data and analysis. All authors have given final approval to the draft.

## Pre-publication history

The pre-publication history for this paper can be accessed here:

http://www.biomedcentral.com/1471-2474/12/122/prepub
